# The distinct epidemic characteristics of HCV co-infection among HIV-1-infected population caused by drug injection and sexual transmission in Yunnan, China

**DOI:** 10.1017/S0950268819001365

**Published:** 2019-09-02

**Authors:** A-Mei Zhang, Ming Yang, Li Gao, Mi Zhang, Lingshuai Jiao, Yue Feng, Xingqi Dong, Xueshan Xia

**Affiliations:** 1 Faculty of Life Science and Technology, Kunming University of Science and Technology, Kunming 650500, China; 2 Yunnan AIDS Care Center (YNACC), Yunnan Provincial Infectious Disease Hospital, Kunming 650500, China

**Keywords:** Biochemical features, genotype, HCV/HIV co-infection

## Abstract

Hepatitis C virus (HCV) infection was frequent in human immunodeficiency virus (HIV) patients in Yunnan province. We studied the epidemic characteristics of HCV in HIV/HCV co-infected patients. Serum from 894 HIV-1 patients was collected, together with basic information and biochemical features. All samples were infected with HIV through injecting drug users (IDUs) and sexual transmission (ST). The *NS5B* gene was amplified and sequenced to affirm HCV genotype. In total, 202 HIV patients were co-infected with HCV, and most (81.19%) of co-infected patients were IDUs. Genotype 3b was predominant (37.62%) in these samples, and its frequency was similar in patients with IDU and ST. The frequencies of genotypes 1a, 1b, 3a, 6a, 6n, 2a and 6u were 3.96%, 16.34%, 23.76%, 6.93%, 10.40%, 0.50% and 0.50%, respectively. However, genotype 3a showed significantly different frequency in HCV patients with IDU and ST (*P* = 0.019). When HCV patients were divided into subgroups, the haemoglobin (HGB) level was significantly higher in patients with genotype 3a than in patients with 3b (*P* = 0.033), 6a (*P* = 0.006) and 6n (*P* = 0.007), respectively. Although no difference existed among HCV subgroups, HIV-viral load was identified to be positively correlated with the HGB level and CD4^+^ cells when dividing HCV/HIV co-infected persons into male and female groups. In conclusion, genotype 3b was the predominant HCV genotype in Yunnan HIV/HCV co-infected persons. The HGB level was higher in patients with genotype 3a than others. HIV-viral load was positively correlated with the HGB level and CD4^+^ cells in the male or female HCV-infected group.

## Introduction

Hepatitis C virus (HCV) infection is still a serious health problem worldwide, especially in the human immunodeficiency virus (HIV)-infected population. The mono-HCV infection has been a handicap for the healthcare system, and about 71 millions of HCV-infected persons developed chronic infection. Approximately 399 thousands of people died because of hepatitis C or diseases caused by HCV infection (cirrhosis and hepatocellular carcinoma (HCC)) each year [1]. HCV infection in the HIV-infected population is more prevalent than the general population, but the occurring rate varied [2]. Until 2017, a total of 36.7 million persons have been infected with HIV worldwide, and about 1 million infected individuals died [3]. Currently, there is no vaccine to prevent persons from HCV and/or HIV infection, so HCV and HIV co-infection needs more attention.

Although direct-acting antiviral therapy is widely used to control HCV prevalence, and about 95% HCV patients could be well cured, the numbers of patients co-infected with HIV and HCV were increased [4] due to shared transmission routes (injection and sexual transmission (ST)) between HCV and HIV infection. HCV infection is more frequent in the HIV-infected population (infectious rate 4.0%) than in other cohorts (infectious rate 2.4%) [2]. It seemed that the elimination of HCV became a serious burden in HIV patients. Patients with various HCV genotypes might express different clinical features and treatment effect, but the HCV genotyping study in HIV-infected persons is rare. Understanding the characteristics of HCV in HIV-infected individuals is necessary to decrease the numbers of the co-infected cohort.

Due to the regional specialty of Yunnan province (neighbour to ‘Golden Triangle’), many injecting drug users (IDUs) existed in Yunnan. One of the most frequent infectious diseases in IDUs was HIV infection; the ratio of HCV is much higher in HIV-infected persons because of their common transmission routes. To investigate the features of HCV genotypes in the HIV-infected population in Yunnan, 202 persons co-infected with HCV and HIV were enrolled for further analysis.

## Materials and methods

### Patients

Basic information and 1 ml serum of 894 inpatient individuals were collected from January 2015 to June 2017 by the doctors in Yunnan Provincial Infectious Disease Hospital. All patients were infected with HIV-1 according to the clinical symptoms and the anti-HIV positive results by using the Anti-HIV ELISA Kit (WANTAI, China), and without any treatment before collection. In total, 359 HIV patients were IDUs, and 535 patients were infected by ST. These patients were from 14 cities of Yunnan province: Kunming (*n* = 62), Xishuangbanna (*n* = 31), Baoshan (*n* = 73), Chuxiong (*n* = 60), Dali (*n* = 7), Dehong (*n* = 61), Honghe (*n* = 100), Linchang (*n* = 101), Nujiang (*n* = 39), Puer (*n* = 90), Qujing (*n* = 55), Wenshan (*n* = 101), Yuxi (*n* = 61) and Zhaotong (*n* = 53). These 14 regions were the main cities in Yunnan province and could represent the cohorts of Yunnan. In total, 567 HIV-infected individuals were male and 327 patients were female. The mean age of all patients was 38.16 ± 10.19 (mean ± s.d.). HCV was identified to be anti-HCV positive in all patients by using the HCV ELISA Kit (ORTHO, USA). Serious liver diseases and other viral infections were absent in all patients. The levels of alanine transaminase (ALT), aspartate transaminase (AST) and haemoglobin (HGB), and the counts of CD4^+^ cells of patients co-infected with HCV and HIV were detected.

### HIV and HCV RNA extraction and genotyping

About 500 µl serum of each patient was collected and used to extract the viral RNA. HIV and HCV RNA were extracted by using the TIANamp virus RNA Kit (TIANGEN, China) according to the manual. The *NS5B* gene of HCV was amplified by using the primers and polymerase chain reaction conditions in our previous study [5]. Then, the products were sequenced by using the Sanger sequencing method.

All sequences of the *NS5B* gene were aligned by using MegAlign software together with 44 reference sequences, which were downloaded from the HCV sequence database (http://hcv.lanl.gov/content/sequence/HCV/ToolsOutline.html). Then, MEGA software was used to construct a neighbour-joining phylogenetic tree and classify samples into different HCV genotypes.

### HIV-viral load quantification

HIV RNA was quantified by using the Quantitative Diagnostic Kit for Human Immunodeficiency Virus (HIV-1) RNA (QIAGEN, China), which was described in previous study [6]. HIV-1-viral load was log 10-transformed for further analysis.

### Data analysis

HCV phylotree was constructed by using MEGA software. The *χ*^2^ test was used to compare the difference between HIV-infected and HIV/HCV co-infected patients. The relationship between HCV genotypes and biochemical features of HCV/HIV co-infected patients was analysed by using the one way analysis of variance test. HIV-viral load was compared between HIV-infected and HIV/HCV co-infected samples by using Student's *t* test (unpaired, two tailed). Correlation matrix was used to analyse the relationship between biochemical features and HIV-viral load in HIV/HCV co-infected patients. The Mann–Whitney *U* test was used to calculate the difference of viral load or biochemical features between different age groups (two tailed). The *P* value less than 0.05 was considered as statistical difference. GraphPad Prism 5 software was used for statistical analysis.

## Results

The age of 88.93% HIV-infected and 97.49% HIV/HCV co-infected patients ranged from 20 to 50 years. Although the distribution showed a statistical difference in subgroups of IDUs and ST patients, the average age of total patients with IDUs and ST was similar (Table 1). The gender ratio (male/female) was significantly different between HIV patients with IDUs (4.79:1) and ST (1.02:1) (*P* < 0.0001). HIV-viral load of each patient was detected. The viral load ranged from 2.98 to 6.15 (log 10-viral load), and the average load of all HIV patients was 4.05 ± 0.67.

**Table 1.** Information of patients infected with HIV and co-infected with HIV/HCV

Subjects	IDUs	ST	*P*-value
Total samples	359 (40.16%)	535 (59.84%)	<0.0001
HCV positive samples	164 (81.19%)	38 (18.81%)	<0.0001
Gender			
Male	297 (82.73%)	270 (50.47%)	<0.0001
Female	62 (17.27%)	265 (49.53%)	
Age^a^			
⩽20	1 (0.28%)	9 (1.68%)	0.103
21–30	25 (6.96%)	156 (29.16%)	<0.0001
31–40	228 (63.51%)	177 (33.08%)	<0.0001
41–50	97 (27.02%)	112 (20.93%)	0.043
51–60	7 (1.95%)	49 (9.16%)	<0.0001
>60	1 (0.28%)	32 (6.73%)	<0.0001
Mean (s.d.)	37.90 ± 5.80	38.33 ± 12.29	0.491
HCV genotype			
1a	5 (3.05%)	3 (7.89%)	0.358
1b	23 (14.02%)	10 (26.32%)	0.109
2a	0 (0%)	1 (2.63%)	0.424
3a	45 (27.44%)	3 (7.89%)	0.019
3b	64 (39.02%)	12 (31.58%)	0.504
6a	12 (7.32%)	2 (5.26%)	0.925
6n	15 (9.15%)	6 (15.79%)	0.361
6u	0 (0%)	1 (2.63%)	0.424
Biochemical index of HIV/HCV co-infected patients			
ALT, IU/L	49.86 ± 40.34	52.29 ± 49.95	0.781
AST, IU/L	58.32 ± 56.25	47.77 ± 36.58	0.157
HGB, g/L	138.8 ± 26.15	137.3 ± 18.65	0.701
CD4^+^, cells/μl	223.5 ± 101.7	268.8 ± 179.7	0.142
HIV-1-viral load	4.10 ± 0.66	4.12 ± 0.68	0.860

a *χ*^2^ test was used to analyse the age difference between the IDU and ST groups. If the number in a group was less than five, Fisher's exact test was used.

A total of 202 HIV patients were identified to be anti-HCV positive by enzyme-linked immunosorbent assay, including 161 males and 41 females. All HCV patients were diagnosed as chronic HCV infection by the doctors according to clinical symptoms, biochemical features and the anti-HCV detection results. Approximately 81.19% of HCV patients were infected by IDUs, and it seemed that drug abuse is the main way for HIV/HCV co-infection. The infectious ratio of HCV had a significant difference between HIV patients with IDUs (45.68%, 164/359) and with ST (7.10%, 38/535) (*P* < 0.0001) in this study (Table 1). Considering the age of the patients might influence the HIV-viral load and biochemical features, all patients were classified into six subgroups according to the age range (i.e. ⩽20, 21–30, 31–40, 41–50, 51–60 and >60) as shown in Table 1. The results showed that the HIV-viral loads of the patients in age 41–50 were statistically higher than that of the patients in age 21–30 (*P* = 0.017), 31–40 (*P* = 0.003) and 51–60 (*P* = 0.020). However, the other biochemical features showed no difference among patients with different age.

**Fig. 1.**
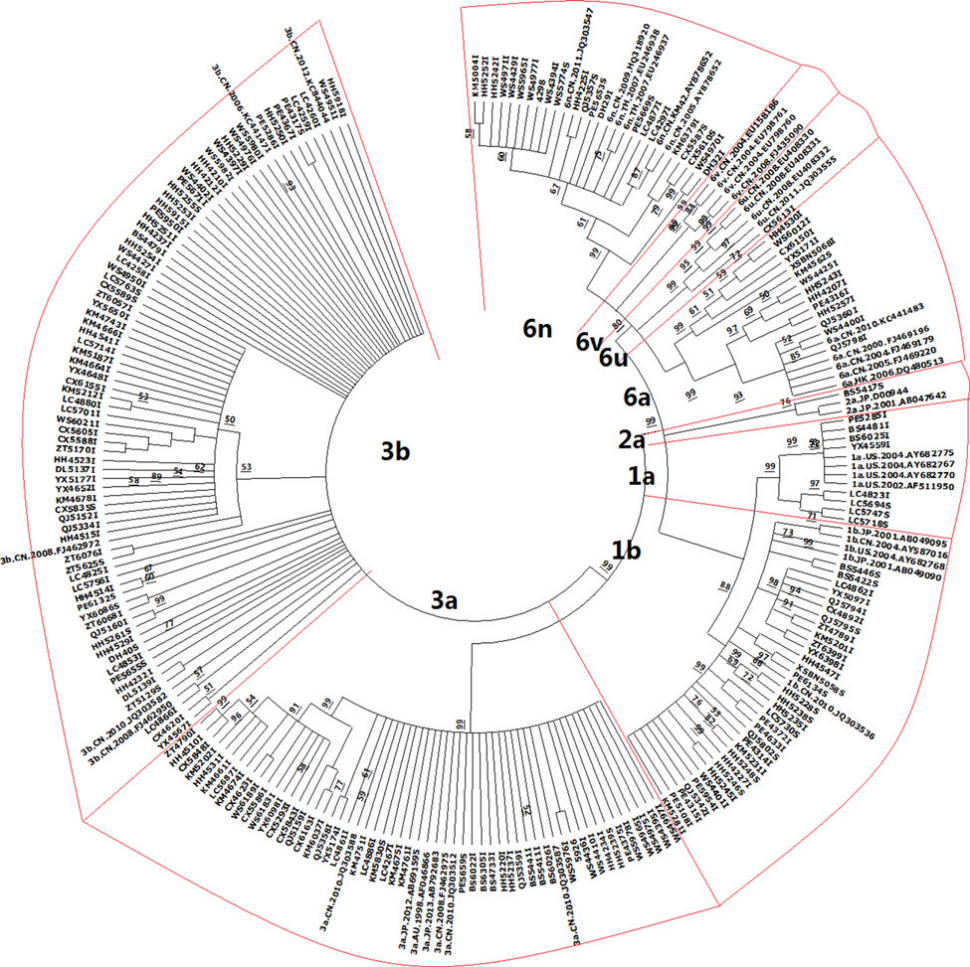
Phylogenetic tree constructed by using 202 HCV strains and 44 reference sequences of HCV. The samples were named by using the cities initials together with numbers: KM for Kunming; XSBN for Xishuangbanna; BS for Baoshan; CX for Chuxiong; DL for Dali; DH for Dehong; HH for Honghe; LC for Linchang; NJ for Nujiang; PE for Puer; QJ for Qujing; WS for Wenshan; YX for Yuxi; ZT for Zhaotong. In total, 44 reference sequences were downloaded from the HCV sequence database (http://hcv.lanl.gov/content/sequence/HCV/ToolsOutline.html). Each branch of HCV genotype was emphasised by a red frame.

After constructing the phylotree according to the reference sequences, HCV genotypes of all samples were identified (Fig. 1). Six genotypes were identified in IDUs and eight genotypes were identified in ST HCV patients, after we compared the accuracy rate of each genotype. The genotype distribution showed a little difference between the two HCV subgroups, but no significance was identified, excluding genotype 3a (*P* = 0.019). The frequency of genotype 3a was 27.44% and 7.89% in HCV patients infected with IDUs and ST, respectively (Table 1).

HIV-viral load was 4.10 ± 0.66 and 4.04 ± 0.67 in HIV patients with and without HCV infection (*P* = 0.218), respectively. HIV-viral load was 4.14 ± 0.66 and 3.97 ± 0.66 in male and female HIV/HCV co-infected patients, respectively. The ALT, AST and HGB levels, the number of CD4^+^ cells and the HIV-viral load were compared between HCV patients infected through IDUs and ST, but no statistical difference was identified (Table 1). Due to only one HCV patient in genotype 2a and 6u group, we neglected these two patients in the following analysis. Biochemical features of HCV patients with genotypes 1a, 1b, 3a, 3b, 6a and 6n were compared. The HGB level showed a significant difference among HCV subgroups with various genotypes (*P* = 0.027), but no other difference was found (Table 2). The HGB level was significantly higher in HCV patients with genotype 3a than patients with genotypes 3b (*P* = 0.033), 6a (*P* = 0.006) and 6n (*P* = 0.007), respectively.

**Table 2.** Biochemical index analysis of HCV patients with different genotypes

Genotype	1a (*n* = 8)	1b (*n* = 33)	3a (*n* = 48)	3b (*n* = 76)	6a (*n* = 14)	6n (*n* = 21)	*P*-value
ALT	40.50 ± 24.95	45.41 ± 45.69	60.54 ± 52.96	50.80 ± 39.29	42.85 ± 22.12	42.42 ± 34.35	0.439
AST	44.25 ± 18.82	50.12 ± 39.16	72.11 ± 87.13	54.60 ± 37.09	52.71 ± 47.99	45.22 ± 28.20	0.298
HGB	136.4 ± 14.02	141.6 ± 22.85	146.1 ± 18.62	137.6 ± 23.13	125.5 ± 36.71	128.3 ± 34.98	0.027
CD4^+^	210.8 ± 72.77	254.3 ± 123.1	230.0 ± 98.48	211.1 ± 123.0	290.9 ± 169.1	230.2 ± 79.38	0.188
HIV-viral load	2.90 ± 0.33	4.15 ± 0.52	4.29 ± 0.79	4.07 ± 0.64	3.70 ± 0.63	4.07 ± 0.66	0.068

The HIV-viral load did not correlate with biochemical features in total HIV/HCV co-infected patients. Particularly, the HGB level and the numbers of CD4^+^ cells were associated with HIV-viral load when we divided all patients into male and female groups (Table 3). Positive correlations were identified between the HIV-viral load and the HGB level (*P* = 0.003 in males, *P* = 0.005 in females) or the CD4^+^ cell counts (*P* < 0.0001 in both males and females). To investigate the effect of age on the correlation between the HIV-viral load and the biochemical features in HIV/HCV co-infected patients, the correlations in different age and gender groups were analysed. The positive correlation (*P* = 0.045) was identified between the HIV-viral load and the AST level in females with age 21–30. However, a negative correlation was found between the HIV-viral load and the CD4^+^ cell counts in males with age 21–30 (Table 3).

**Table 3.** Correlation between biochemical features and HIV-viral load in HIV/HCV co-infected patients

Biochemical features	ALT	AST	HGB	CD4^+^
Total				
*r*	−0.052	−0.073	−0.092	−0.111
*P*-value	0.469	0.302	0.195	0.117
Male				
*r*	0.001	0.020	0.236	0.904
*P*-value	0.986	0.804	0.003	<0.0001
Female				
*r*	−0.314	−0.244	0.427	0.845
*P*-value	0.045	0.125	0.005	<0.0001
Age^a^				
21–30 Male	*r* = 0.094 (*P* = 0.772)	*r* = 0.130 (*P* = 0.688)	*r* = −0.234 (*P* = 0.465)	*r* = −0.610 (*P* = 0.035)
21–30 Female	*r* = 0.761 (*P* = 0.079)	*r* = 0.820 (*P* = 0.045)	*r* = −0.405 (*P* = 0.425)	*r* = −0.545 (*P* = 0.263)
31–40 Male	*r* = −0.114 (*P* = 0.264)	*r* = −0.107 (*P* = 0.293)	*r* = −0.082 (*P* = 0.419)	*r* = −0.089 (*P* = 0.385)
31–40 Female	*r* = −0.111 (*P* = 0.596)	*r* = −0.297 (*P* = 0.150)	*r* = −0.205 (*P* = 0.326)	*r* = −0.360 (*P* = 0.077)
41–50 Male	*r* = −0.165 (*P* = 0.256)	*r* = 0.005 (*P* = 0.975)	*r* = −0.075 (*P* = 0.611)	*r* = 0.020 (*P* = 0.892)
41–50 Female	*r* = 0.496 (*P* = 0.174)	*r* = 0.232 (*P* = 0.549)	*r* = 0.035 (*P* = 0.928)	*r* = −0.015 (*P* = 0.969)

a Because only one and two patients belonged to groups with age ⩽20 and 51–60, respectively, and no patients belonged to group with age >60, the correlation between HIV-viral load and biochemical features of patients was not analysed.

## Discussion

Due to the lack of vaccine and HCV gene mutations, the protection and treatment of HCV infection need further study. Yunnan province is located in Southwest of China, neighbours the ‘Golden Triangle’. Thus, HIV-infected cohort is large in Yunnan. Drug injection and sexual behaviour are two main transmission routes of HIV infection, and HCV was one of the most frequent viruses co-infected with HIV [5, 7]. The HCV infectious rate is much higher in the HIV-infected population than in the general population [8, 9]. However, most of the HCV genotype analysis focused on IDU patients. IDUs seemed the main transmission route of HCV infection, with infectious rate ranging from 22% to 95% [10]. However, HCV-pooled prevalence was a little lower in Yunnan IDUs (76.6%) than in Guangxi (83.8%), Hubei (91.8%) and Hunan (78.7%) IDUs in Xia *et al*. [10]. In this study, we find that the HCV co-infected rate in HIV patients was 22.60% (202/894). It should be noted that the HCV co-infected rate was significantly higher in IDUs (45.68%) than in ST (7.10%) HIV patients, thus the low co-infected rate of HIV and HCV in this study was caused by ST. It was necessary to analyse the epidemic characteristic of HCV infection in HIV patients.

When the HCV genotypes in this study were compared to previously reported data in Yunnan [5, 11–13], HCV genotype 1b showed a difference between the two cohorts. The ratio of genotype 1b was 16.33% and 15.37% in this study and the reported data, respectively. Although the infectious rate of genotype 1b seemed similar, statistical analysis showed a statistical difference (*P* = 0.046). Because all reported HCV patients were IDUs with HIV, which suggested the difference might be related to different transmission routes. No significant difference was identified when comparing genotype 1b between the reported data and HCV with IDUs in this study. Although the ratio of genotype 1b was higher in patients with ST (26.32%, 10/38) than IDUs (15.08%, 114/756), no statistical difference existed. This result might be due to the small sample size of HIV/HCV patients with ST, thus enlarging the cohort for further analysis was necessary.

HCV genotypes showed a different distribution among various provinces in China. Genotype 3 (including 3a and 3b) occupied 61.39% (124/202) of total HCV-infected persons in this study, and this ratio is similar to the reported ratio (53.54%, 317/592) [5, 11–13]. Genotype 3, especially genotype 3b, was the dominant HCV genotype in the HCV population in Yunnan, which was considered to be originating from Vietnam. Genotype 6a was the predominant HCV genotype in many regions in China, such as Hubei, Guangxi and Guangzhou [14–17]. Genotypes 3a and 1b were prevalent in Ningxia [8] and Jiangsu [18], respectively. It suggested that further analysis of the HCV genotypes in various provinces in China and investigating their epidemic features were necessary, which will benefit to control the HCV prevalence in the Chinese population.

It was the first time to analyse the biochemical characteristics in HIV/HCV co-infected persons in Yunnan, China. Although the ALT and AST levels showed no difference between HCV patients by IDUs or ST with different genotypes, the HGB level was significantly higher in HCV patients with genotype 3a than in patients with genotypes 3b, 6a and 6n, respectively (Table 2). Wei *et al*. reported that the serum iron level was a risk factor for hepatitis B virus (HBV)-related HCC, and it was also positively correlated with the HGB level [19]. It seemed that the HGB level also correlated with HBV-related HCC. The function of HGB was containing and transporting oxygen in red blood cells. Thus, we assumed that the HGB level might correlate with HCV infection in different genotypes by the serum iron level, but it still needs to be verified.

A special phenomenon was expressed when we analysed the correlation between HIV-viral load and biochemical features of the HIV/HCV co-infected patients. Although all biochemical characteristics showed no correlation with HIV-viral load of total patients, the ALT, HGB levels and CD4^+^ cells significantly correlated with HIV-viral load in male and female groups (Table 3). The ALT level negatively correlated with HIV-viral load in female HIV/HCV co-infected patients, and the HGB and CD4^+^ cells were positively correlated with HIV-viral load in male and female patients, respectively. It seems that these correlations were not caused by age difference (Table 3). Caffeine consumption could lead to higher CD4^+^ cells and lower HIV-viral load [20], but no analysis between CD4^+^ cells and lower HIV-viral load was performed. It needed us to investigate more factors which play roles in the correlation between HIV-viral load and CD4^+^ cells, such as host genetic polymorphisms, variety of drug or other pathogenic microorganism infections.

In summary, we studied the HCV genotype distribution and the epidemic characteristics of HIV/HCV co-infected persons in Yunnan, China. The results showed that genotype 3b was the predominant genotype in Yunnan. We firstly identified that the HGB level was significantly different among patients with different HCV genotypes, and HIV-viral load correlated with biochemical features of HIV/HCV co-infected persons.
